# pH- and Thermo-Responsive Water-Soluble Smart Polyion Complex (PIC) Vesicle with Polyampholyte Shells

**DOI:** 10.3390/polym14091659

**Published:** 2022-04-20

**Authors:** Thu Thao Pham, Tien Duc Pham, Shin-ichi Yusa

**Affiliations:** 1 Department of Applied Chemistry, Graduate School of Engineering, University of Hyogo, 2167 Shosha, Himeji 671-2280, Hyogo, Japan; phamthuthao.hus@gmail.com; 2 Faculty of Chemistry, University of Science, Vietnam National University, Hanoi, 19 Le Thanh Tong, Hoan Kiem, Hanoi 100000, Vietnam; tienduchphn@gmail.com

**Keywords:** polyion complex, electrostatic interaction, oppositely charged polyelectrolyte, pH-responsive, UCST behavior, polyampholyte

## Abstract

A diblock copolymer (P(VBTAC/NaSS)_17_-*b*-PAPTAC_50_; P(VS)_17_A_50_) composed of amphoteric random copolymer, poly(vinylbenzyl trimethylammonium chloride-*co*-sodium *p*-styrensunfonate) (P(VBTAC/NaSS); P(VS)) and cationic poly(3-(acrylamidopropyl) trimethylammonium chloride) (PAPTAC; A) block, and poly(acrylic acid) (PAAc_49_) were prepared via a reversible addition−fragmentation chain transfer radical polymerization. Scrips V, S, and A represent VBTAC, NaSS, and PAPTAC blocks, respectively. Water-soluble polyion complex (PIC) vesicles were formed by mixing P(VS)_17_A_50_ and PAAc_49_ in water under basic conditions through electrostatic interactions between the cationic PAPTAC block and PAA_c49_ with the deprotonated pendant carboxylate anions. The PIC vesicle collapsed under an acidic medium because the pendant carboxylate anions in PAAc_49_ were protonated to delete the anionic charges. The PIC vesicle comprises an ionic PAPTAC/PAAc membrane coated with amphoteric random copolymer P(VS)_17_ shells. The PIC vesicle showed upper critical solution temperature (UCST) behavior in aqueous solutions because of the P(VS)_17_ shells. The pH- and thermo-responsive behavior of the PIC vesicle were studied using ^1^H NMR, static and dynamic light scattering, and percent transmittance measurements. When the ratio of the oppositely charged polymers in PAPTAC/PAAc was equal, the size and light scattering intensity of the PIC vesicle reached maximum values. The hydrophilic guest molecules can be encapsulated into the PIC vesicle at the base medium and released under acidic conditions. It is expected that the PIC vesicles will be applied as a smart drug delivery system.

## 1. Introduction

Owing to electrostatic interactions between cationic and anionic units, oppositely charged polyelectrolytes in water form a polyion complex (PIC) or polyelectrolytes complex [1,2]. Furthermore, PIC micelles with a PIC core and hydrophilic noncharged shells can be prepared without using an organic solvent in water [3,4]. Smart PIC aggregates have single- or multi-responsive properties against external stimuli, such as pH, temperature, and light irradiation. They show substantial changes in their properties with a slight change in the surrounding environments [5]. The PIC aggregates have attracted huge interest from many researchers because of their advantages. The PIC aggregates are expected to meet some drug delivery system (DDS) assignments, such as the targeted and controlled release of the drug. The pH of the medium is an important factor in the controlled release of a DDS. The microenvironment of cancer tumors is acidic, whereas it is nearly neutral for normal cells [6]. The change in the pH of the environment results in an ideal pH-responsive DDS carrier, which can be applied in cancer treatments. Therefore, many PIC aggregate systems, which target the release of drugs in response to the acidity of the surroundings, have been reported [7,8,9,10,11,12]. Water-soluble pH-responsive PIC vesicles covered with biocompatible poly(2-(methacryloyloxy)ethyl phosphorylcholine) (PMPC) shells have also been reported [3]. The PIC vesicle was coassembled via electrostatic interactions between PMAPTAC and ionized PAaH under a basic medium by mixing oppositely charged diblock copolymers consisting of PMPC and cationic poly(3-(methacrylamidopropyl) trimethylammonium chloride) (PMAPTAC), or anionic poly(sodium 6-acrylamidohexanoate) (PAaH) blocks. The hydrophilic guest molecule, Texas red-labeled dextran, was encapsulated into the inner water phase of the PIC vesicle. Under the acidic medium, PAaH was protonated to become noncharged resulting in a dissociation of the PIC vesicle, where the guest molecule was released. Therefore, the PIC vesicle is expected to effectively target the drug in the cytoplasm.

Similarly, the environment’s temperature is a widely used parameter in the controlled release of drugs [13,14,15,16,17]. Matsuoka et al. [13] reported on thermo-responsive PIC aggregates. Diblock copolymers composed of poly(sulfopropyl dimethylammonium propylacrylamide) (PSPP) and cationic PMAPTAC or anionic poly(sodium *p*-styrenesulfonate) (PNaSS) were mixed in water to form PIC micelles with a PMAPTAC/PNaSS core and PSPP shells. The PIC micelles showed an upper critical solution temperature (UCST) caused by the PSPP shells. They formed large aggregates of micelles below the phase transition temperature of the PSPP shells owing to the PSPP chain shrinkage. The UCST behavior of the PIC aggregates can be controlled using the polymer concentration and the degree of polymerization (DP) of the PSPP chains. Furthermore, some other PIC systems have been fabricated with the target to release a drug in response to environments containing glucose to deal with diabetes [18,19] or to use light irradiation to control the release of the drug [20,21].

These single stimulus-responsive PIC aggregates have been studied and applied. Besides stimuli response, PIC aggregates have shown other outstanding advantages [22,23,24,25,26,27]. For instance, dual pH- and thermo-responsive PIC micelles were prepared from a co-assembly of oppositely charged diblock copolymers, poly(*N*-methyl-2-vinyl pyridinium iodide)-*block*-poly(ethylene oxide) (P2MVP-*b*-PEO) and poly(acrylic acid)-*block*-poly(isopropyl acrylamide) (PAAc-*b*-PNIPAM) in water [27]. Under a basic medium at 25 °C, the PIC micelles were formed owing to the electrostatic interaction between the PAAc and P2MPV blocks with the PAAc/P2MVP core and hydrophilic mixed PNIPAM/PEG shells. However, under an acidic medium, the size of the aggregates increased, which can be attributed to the aggregation formed by hydrogen bonding between PAAc and PNIPAM/PEO. At high concentrations, the core–shell structure of PIC micelles switches to core–shell–corona (onion type) with a PNIPAM core, covered with a mixed P2MVP/PAAc shell, and a PEO outer corona at 60 °C.

Polyampholytes or amphoteric random copolymers are polymers that consist of cationic and anionic units in their structure [28,29,30]. Amphoteric random copolymers have no charge, which is comparable to the balance between oppositely charged groups since they contain both pendant cationic and anionic groups [31,32]. Therefore, to avoid complex precipitation, the polyampholytes can be used as a hydrophilic nonionic block. Furthermore, some amphoteric polymers show thermo-responsive and protein antifouling properties. Yusa et al. [28] reported that amphoteric diblock copolymers from poly(2-acrylamido-2-methylpropanesulfonic acid sodium salt) (PAMPS) and poly(3-(acrylamido)propyl trimethylammonium chloride) (PAPTAC) synthesized via a reversible addition−fragmentation chain transfer (RAFT) radical polymerization. The polymer exhibited a lower critical solution temperature (LCST) behavior due to the hydrogen-bonding interactions of the pendant amide groups and water molecules. The thermo-responsive behavior of the polymers can be controlled by adjusting the NaCl concentration. Meanwhile, P(APMPS/APTAC), a random copolymer prepared by RAFT with the same content as APMPS and APTAC, showed an ability in protein antifouling [33]. The PIC micelle consists of amphoteric random copolymer P(AMPS/APTAC) shells that show no interaction with proteins. Furthermore, a UCST-type thermo-responsive amphoteric random copolymer (P(VBTAC/NaSS)) composed of the pendant quaternary ammonium group in vinylbenzyl trimethylammonium chloride (VBTAC) and pendant sulfonate in NaSS with the same content of each monomer, was prepared via RAFT polymerization [34]. P(VBTAC/NaSS) showed the UCST behavior caused by electrostatic interactions between PVBTAC and PNaSS. The UCST behavior was affected by DP and concentrations of P(VBTAC/NaSS).

**Scheme 1:**
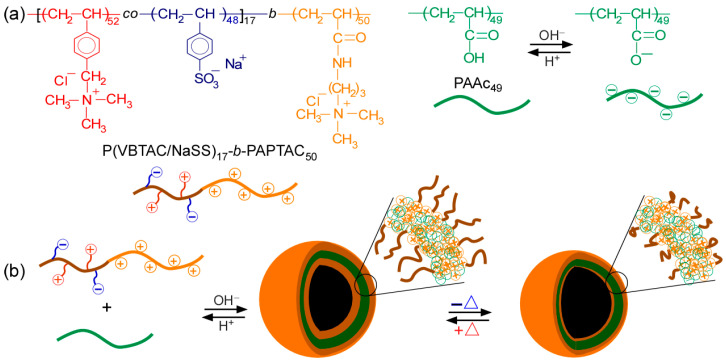
(**a**) Chemical structures of P(VBTAC/NaSS)_17_-*b*-PAPTAC_50_ ((P(VS)_17_A_50_) and PAAc_49_, and (**b**) conceptual formation of the PIC vesicle consist of P(VS)_17_A_50_ and PAAc_49_.

Based on prior studies, we designed pH- and thermos-responsive PIC micelles. The interaction of the PIC vesicle with the hydrophobic surface may be controlled by temperature if the PIC vesicle surface hydrophobicity is changed by heating. For example, if the PIC vesicle surface changes to be hydrophobic with cooling, the interactions increase between the PIC vesicle and cell. Furthermore, if the PIC vesicle is dissociated in response to pH, the encapsulated guest molecules are released by pH change. Particularly, cancer tissue is more acidic than healthy tissue, so designing a pH-responsive PIC vesicle is useful for cancer treatment. In this study, we have prepared a diblock copolymer (P(VS)_17_A_50_) consisting of polyampholyte (P(VBTAC/NaSS)_17_; P(VS)_17_) block and a cationic PAPTAC_50_ (A_50_) block (Scheme 1). Furthermore, the PIC aggregates were prepared by mixing P(VS)_17_A_50_ and polyacrylic acid (PAAc_49_) in a basic medium because of electrostatic interactions between the cationic PAPTAC block and deprotonated anionic PAAc_49_. The PIC aggregate had a vesicle structure, which confirmed that the PIC aggregate could encapsulate a hydrophilic guest molecule into the hollow core. PAAc indicated pH-responsive behavior caused by protonation and the pendant carboxyl groups [35,36,37]. The PAAc pendant carboxyl groups are protonated and neutralized under acidic conditions, leading to the dissociation of the PIC vesicles. The PIC vesicle was formed from the PAPTAC/PAAc membrane, covered with the amphoteric random copolymer P(VS)_17_ shells. In this study, to synthesize P(VS)_17_, equimolar amounts of styrene-type cationic VBTAC and anionic NaSS were randomly copolymerized via RAFT. Then, the cationic APTAC monomer was polymerized to synthesize P(SV)_17_A_50_. Furthermore, PAAc_49_ was also synthesized via RAFT. Polymers were characterized with NMR and gel-permeation chromatography (GPC) measurements. The pH- and thermo-responsive properties of the PIC vesicle were studied with percent transmittance, dynamic and static light scattering, zeta-potential, and NMR measurements. Additionally, since the P(SV)_17_ polyampholyte shells exhibited the UCST behavior, controlling the interaction with proteins and cells in response to temperature may be possible. Furthermore, the P(SV)_17_ polyampholyte can suppress protein adsorption. Therefore, the PIC vesicles are expected to modify the surfaces of medical devices.

## 2. Materials and Methods

### 2.1. Materials

Vinylbenzyl trimethylammonium chloride (VBTAC, 99%) methoxy poly(ethylene glycol) labeled pyrene (PEG-Py, *M*_n_ = 2000 g/mol) was purchased from Sigma-Aldrich (St. Louis, MO, USA), sodium p-styrenesulfonate (NaSS, 98%) from Tokyo Chemical Industry (Tokyo, Japan), and 4,4-azobis(4-cyanopentanoic acid) (V-501, 98%) and 2,2-azobisisobutyronitrile (AIBN, 98%) from Wako Pure Chemical (Osaka, Japan) were used without further purification. Acrylic acid (AAc, 98%) from Wako Pure Chemical (Osaka, Japan) was distilled under reduced pressure. 4-Cyanopentanoic acid dithiobenzoate (CPD) was synthesized using the previously reported method [38]. The water was purified using an ion-exchange column system.

### 2.2. Synthesis of P(VS)_17_ Random Copolymer

First, amphoteric random copolymer (P(VS)_17_) was prepared via RAFT polymerization in accordance with the previous report [34]. VTBAC (3.18 g, 15.0 mmol), NaSS (3.09 g, 15.0 mmol), CPD (419 mg, 1.50 mmol), and V-501 (210 mg, 0.749 mmol) were dissolved in 30 mL of mixed solvent of 1.2 M NaCl aqueous solution and MeOH (9/1, *v/v*) with the feed molar ratio of [VBTAC]/[NaSS]/[CPD]/[V-501] = 10/10/1/0.5. The mixture was then, degassed by purging with Ar gas for 30 min. Polymerization occurred at 70 °C for 5 h. After polymerization, ^1^H NMR spectra were used to calculate the polymerization conversion of VBTAC and NaSS (conversion = 84.5%). The reaction mixture was dialyzed against 1.2-M NaCl aqueous solution for two days and pure water for one day and collected using a freeze-drying method (4.69 g, 67.9%). The theoretical DP(theo)) and number-average molecular weight (*M*_n_(theo)) were 17 and 3.83 × 10^3^ g/mol. The VBTAC and NaSS contents were determined using ^1^H NMR spectra of P(VS)_17_ and found to be 51.9 and 48.1 mol%, respectively.

### 2.3. P(VBTAC/NaSS)_17_-b-PAPTAC_50_

The diblock copolymer was prepared via RAFT polymerization using P(VS)_17_ as a macro-CTA. P(VS)_17_ (539 mg, 0.141 mmol), APTAC (1.46 g, 7.06 mmol), and V-501 (15.7 mg, 0.06 mmol) with a feed molar ratio of [P(VS)_17_]/[APTAC]/[AIBN] = 1/50/0.4 were dissolved in water (7.0 mL). The mixture was degassed by purging with Ar gas for 30 min. Polymerization occurred at 70 °C for 24 h. The polymerization conversion was calculated from ^1^H NMR integral intensity ratio of the vinyl protons and phenyl protons to 5.6 and 6.4–7.6 ppm, respectively, (conversion = 98.4%). Then, the reaction mixture was dialyzed against pure water for 5 days and collected using a freeze-drying method (1.55 g, 77.5%). From ^1^H NMR measurement, the DP(NMR)) of the APTAC block was estimated to be 50. The number-average molecular weight (*M*_n_(GPC)) and polydispersity index (*M*_w_/*M*_n_) of P(VS)_17_A_50_ were estimated to be 5.70 × 10^4^ g/mol and 1.04, respectively, based on the GPC measurement.

### 2.4. Synthesis of PAAc_49_

RAFT polymerization was used to prepare poly(acrylic acid) (PAAc_49_). AAc (7.20 g, 100 mmol), CPD (280 mg, 0.99 mg), and AIBN (65.7 mg, 0.40 mmol) with a feed molar ratio of [AAc]/[CPD]/[AIBN] = 100/1/0.4 were dissolved in methanol (100 mL). The mixture was degassed by purging with Ar gas for 30 min. Polymerization occurred at 60 °C for 47 h. From the ^1^H NMR integral intensity ratio, the polymerization conversion of the vinyl proton signal at 5.8 ppm and terminal phenyl group protons at 7.3–7.9 ppm (conversion = 36.3%) was estimated. Then, the reaction mixture was dialyzed against pure water for one week and collected using a freeze-drying method (0.97 g, 12.9%). From ^1^H NMR measurement the DP(NMR) of PAAc_49_ was calculated to be 49. From GPC measurement, the *M*_n_(GPC) and *M*_w_/*M*_n_ of P(VS)_17_A_50_ were 1.46 × 10^4^ g/mol and 1.07, respectively.

### 2.5. Preparation of PIC Vesicles

P(VS)_17_A_50_ and PAAc_49_ were individually dissolved in water at *C*_p_ = 1.0 g/L as stock solutions to prepare the PIC vesicles. The PIC vesicles were fabricated by mixing the cationic P(VS)_17_A_50_ solution with the anionic PAAc_49_ solution while stirring for 5 min at room temperature. The pH of the mixed solution was adjusted using 0.1-M NaOH and 0.1-M HCl aqueous solutions, then left for one day to obtain an equilibrium state. The mole fraction of positively charged (*f*^+^ = [APTAC]/([APTAC] + [PAAc]) in the PIC vesicle solution corresponds to the mixing ratio of the opposite polymers 

### 2.6. Encapsulation of PEG-Py

PEG-Py (0.01 g/L) aqueous solution was prepared. Then, P(VS)_17_A_50_ (0.1 g/L) and PAAc_49_ (0.1 g/L) were separately dissolved in the PEG-Py aqueous solution. Subsequently, the P(VS)_17_A_50_ aqueous solution was added to the PAAc_49_ aqueous solution with *f*^+^ = 0.5 and stirred for 5 min at room temperature. The mixed solution was adjusted to pH 10 using NaOH solution and then dialyzed for 20 days against an aqueous solution at pH 10 using a Harvard Apparatus (Holliston, MA, USA) polycarbonate membrane with 10-nm pores. The solvent was replaced every 12 h to remove the unbound PEG-Py molecules. A PEG-Py aqueous solution (0.01 g/L) without a PIC vesicle was dialyzed with the same procedure. Fluorescence spectroscopy measurements for PEG-Py in the presence and absence of the PIC vesicles were performed to detect PEG-Py content in the solution after dialysis. The loading capacity (LC) and loading efficiency (LE) were calculated according to the following Equations [39]:

(1) $$\mathrm{LE}\;\left(\%\right)=\frac{\mathrm{Weight}\;\mathrm{of}\;\mathrm{encapsulated}\;\mathrm{PEG}-\mathrm{Py}}{\mathrm{Weight}\;\mathrm{of}\;\mathrm{total}\;\mathrm{PEG}-\mathrm{Py}}\;\times \;100,$$

(2) $$\mathrm{LC}\;\left(\%\right)=\frac{\mathrm{Weight}\;\mathrm{of}\;\mathrm{encapsulated}\;\mathrm{PEG}-\mathrm{Py}}{\mathrm{Weight}\;\mathrm{of}\;\mathrm{the}\;\mathrm{PIC}\;\mathrm{aggregate}}\;\times \;100.\;$$

### 2.7. Measurements

^1^H NMR measurements were performed to determine the complex characteristics of the polymer and polyion complex using a JEOL (Tokyo, Japan) JNM-ECZ 400 MHz NMR. The sample solutions were prepared in D_2_O and adjusted with NaOD or DCl solutions to desired pH. The standard pulse program, stebpgp1s19, used a stimulated echo, bipolar gradient pulse, and one spoil gradient with 3-9-19 pulse sequence (WATERGATE) solvent suppression of the water signal. The GPC measurements were used to determine the number-average molecular weight (*M*_n_(GPC)) and polydispersity index (*M*_w_/*M*_n_). For cationic polymer, P(VS)_17_A_50_, a mixture of Na_2_SO_4_ (0.3 M) and CH_3_COOH (0.5 M) aqueous solution was used as a cationic eluent. P(VS)_17_A_50_ signal was detected using Jasco (Tokyo, Japan) RI-2031 Plus refractive index (RI) detector equipped with a PU-2080 Plus column operating at 40 °C. Using a calibrated curve of standard poly(2-vinylpyridine), the values of *M*_n_(GPC) and *M*_w_/*M*_n_ for P(VS)_17_A_50_ were estimated. For anionic homopolymer PAAc_49_, GPC spectra were obtained from Tosoh RI-8020 RI working at 40 °C with a Shodex (Tokyo, Japan) GF-7M column, Tosoh (Yamaguchi, Japan) DP-8020 pump and a mixture of phosphate buffer (50 mM) at pH 9 and acetonitrile (9/1, *v/v*) as an anionic eluent. Using a calibration made from standard PNaSS, the values of *M*_n_ and *M*_w_/*M*_n_ for polymers were estimated. Fourier transform infrared (FTIR) spectra were obtained using a Jasco FT/IR-4200 spectrometer. The PIC vesicle structure was confirmed via transmission electron microscopy (TEM) observation using a JEOL (Tokyo, Japan) JEM-2100. The PIC vesicle aqueous solutions with *f*^+^ = 0.5 and *C*_p_ = 0.1 g/L at pH 10 were prepared. Then, a drop of the sample solution was put on a copper grid coated with thin films of Formvar. The oversupply sample solution was filtered using filter paper. The next step was to stain with sodium phosphotungstate and dry under a vacuum for one night. The hydrodynamic radius (*R*_h_), light scattering intensity (LSI), and zeta-potential of polymers and PIC vesicles were calculated using a Malvern Nano ZS with He–Ne laser (4 mW at 632.8 nm) from Malvern (Kobe, Japan). Before taking the measurements, the sample solutions were filtered using a 0.45-μm pore size membrane filter. Furthermore, to estimate the weight-average molecular weight (*M*_w_), a *z*-average radius of gyration (*R*_g_), and the second viral coefficient (*A*_2_) values of the polymers and PIC, SLS measurements were performed using the Otsuka Electronics Photal (Osaka, Japan) SLS-6500 with a He–Ne laser (4 mW at 632.8 nm) as a light source working at 25 °C. An Otsuka Electronics Photal DRM-3000 (Osaka, Japan) differential refract meter was used to calculate d*n*/d*C*_p_ values at 633 nm. Percentage transmittance (%*T*) was measured on a Jasco (Tokyo, Japan) V-730 UV-vis spectrophotometer equipped with a temperature control system. Fluorescence spectra for PEG-Py were measured using a Hitachi (Tokyo, Japan) F-2500 fluorescence spectrometer. The excitation and emission slit widths were kept constantly at 20 and 2.5 nm, respectively.

## 3. Results

### 3.1. Preparation of P(VS)_17_A_50_ and PAAc_49_

**Figure 1:**
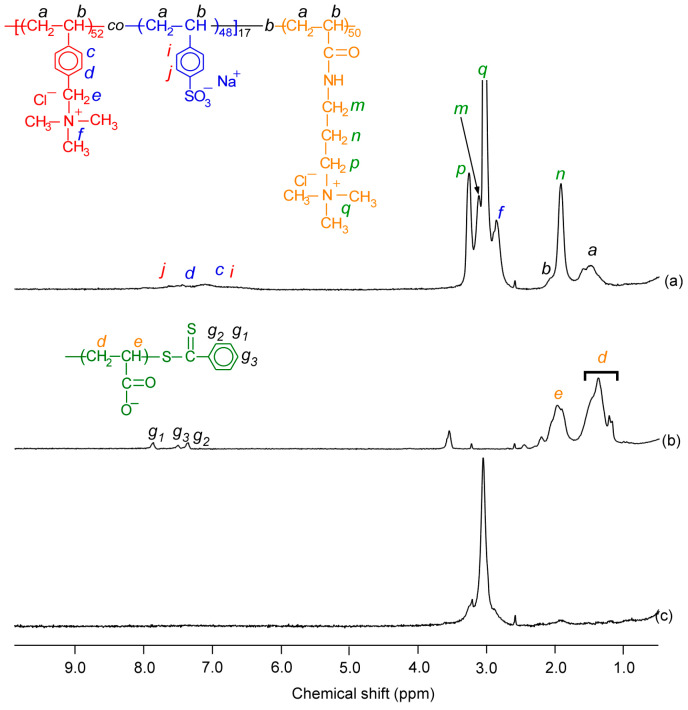
^1^H NMR with water suppression spectra for P(VS)_17_A_50_ (**a**), PAAc_49_ (**b**), and the P(VS)_17_A_50_/PAAc_49_ PIC vesicle with *f*^+^ = 0.5 (**c**) at *C*_p_ = 2.0 g/L in D_2_O at pH 10 at 25 °C.

All polymers in this study were prepared via normal RAFT polymerization. An amphoteric random copolymer comprising cationic VBVTAC and anionic NaSS was prepared via RAFT radical polymerization. According to ^1^H NMR, the total conversion (*p*) of VBTAC and NaSS was estimated to be 84.5%. The theoretical DP(theo) and theoretical number-average molecular weight (*M*_n_(theo)) of P(VS)_17_ were calculated using *p* and the following equation:

(3) $$\mathrm{DP}\left(\mathrm{theo}\right)=\frac{{\left[M\right]}_{0}}{{\left[\mathrm{CTA}\right]}_{0}}\;\times \;\frac{p}{100\;}\;$$

(4) $$M_{n}{(\mathrm{theo})=\mathrm{DP}}_{\mathrm{theo}}{\;\times \;M}_{m}{+M}_{\mathrm{CTA}}$$

where, [M]_0_ and [CTA]_0_ are the initial concentrations of the monomer and CTA, respectively, and M_m_ and M_CTA_ are the molecular weights of the monomer and CTA, respectively. The DP(theo) and *M*_n_(theo) for P(VS)_17_ were calculated to be 17 and 3.83 × 10^3^ g/mol, respectively. The composition of P(VS)_17_ was estimated from ^1^H NMR in D_2_O containing 1.2 M NaCl at 70 °C (Figure S1). The main chain proton signals were observed at 1.5–2.1 ppm. The pendent methyl protons of the VTBAC unit were observed at 2.9 ppm. The pendant phenyl protons of the VBTAC and NaSS units are presented as 6.4–7.6 ppm. The VBTAC content in the P(VS)_17_ block was calculated to be 51.9 mol% based on the integral intensities between the pendant phenyl and VBTAC methyl protons. The DP(NMR) for the PAPTAC block in P(VS)_17_A_50_ was 50, as estimated from the integral intensity of the methylene proton from the PAPTAC unit at 1.6 ppm compared with that for the phenyl protons of VBTAC and NaSS at 6.4–7.6 ppm (Figure 1a). PAAc_49_ has a DP(NMR) of 49, estimated from the ratio of integral intensities between main chain protons at 1.5–2.3 ppm and terminal phenyl protons at 7.3–7.9 ppm, derived from the CTA fragment (Figure 1b). The *M*_n_(NMR) and *M*_n_(theo) values for P(VS)_17_A_50_ were close. The *M*_w_/*M*_n_ values estimated from GPC for P(VS)_17_A_50_ and PAAc_49_ (Figure S2) were 1.04 and 1.07, respectively, indicating that the polymers showed well-controlled structures. The DP, *M*_n_, and *M*_w_/*M*_n_ values for all polymers are summarized in Table 1. P(VS)_17_ could not be measured using GPC because it cannot dissolve in the eluent. FTIR spectrum was measured for P(VS)_17_A_50_. The pendant carbonyl signal can be seen at 1700 cm^−1^ (Figure S3).

### 3.2. Preparation and Characterization of PIC Vesicles

The PIC vesicle was prepared by mixing P(VS)_17_A_50_ and PAAc_49_ with *f*^+^ = 0.5 at *C*_p_ = 2.0 g/L in D_2_O at pH 10 for the ^1^H NMR measurement (Figure 1c). For DLS measurements, the PIC vesicle was prepared at 0.1 g/L; however, the *C*_p_ was too low for NMR measurements so the PIC vesicle was prepared at *C*_p_ = 2.0 g/L. After mixing, the signals from the PAPTAC_50_ block and PAAc_49_ disappeared. The VBTAC unit’s methylene proton signal remained at 2.9 ppm, showing that the PAPTAC block and PAA_c49_ interacted to create the vesicle structure’s membrane and P(VS)_17_ produced shells.

**Figure 2:**
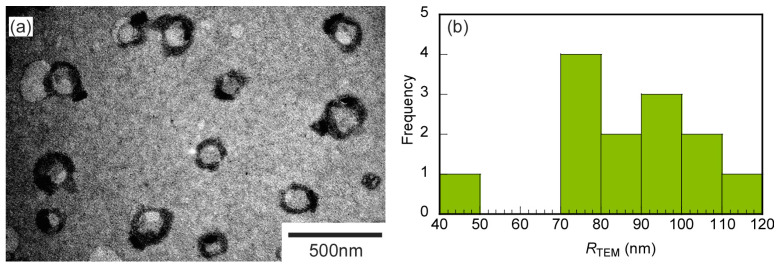
(**a**) TEM image and (**b**) histogram of particle size distribution for the P(VS)_17_A_50_/PAAc_49_ PIC vesicle with *f*^+^ = 0.5 at *C*_p_ = 0.1 g/L in water at pH 10.

TEM was conducted to confirm the PIC aggregate shape. The TEM image was obtained for the stoichiometrically charge neutralized mixture of P(VS)_17_A_50_ and PAAc_49_ at pH 10 (Figure 2). The particles had a spherical shape with a contrasting white center and black edge, indicating a vesicle structure in the PIC aggregates. The average radius obtained from the TEM observations was 86.2 ± 17.3 nm, close to the hydrodynamic radius (*R*_h_) estimated from the dynamic light scattering measurements (*R*_h_ = 86.6 nm). During TEM observation, the sample shrank due to the high vacuum conditions. The PIC vesicles collapsed as they shrank and stuck to the TEM grid membrane. At that time, the PIC vesicles expanded. Therefore, the size obtained from the TEM observation did not decrease much compared to the *R*_h_ value.

**Figure 3:**
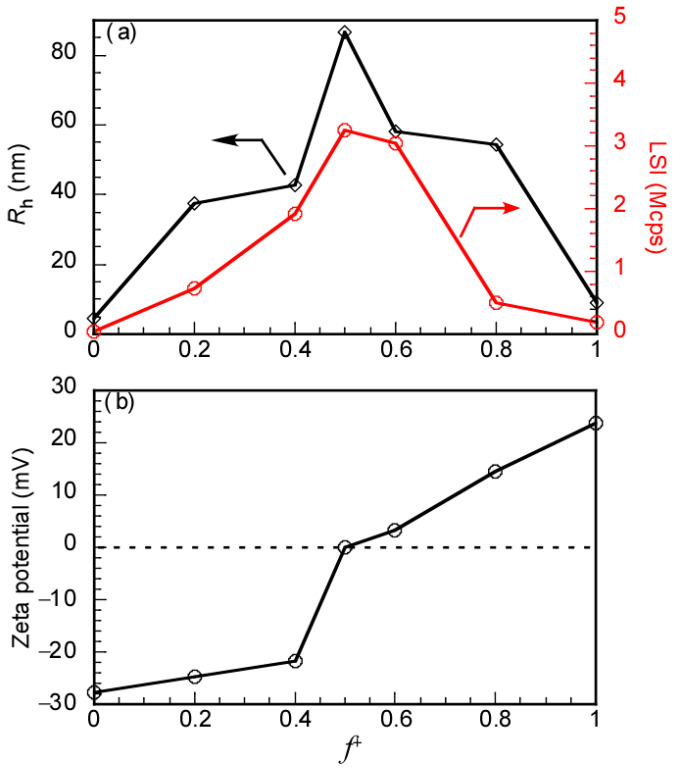
(**a**) Hydrodynamic radius (*R*_h_, diamond) and light scattering intensity (LSI, circle) and (**b**) zeta-potential for the P(VS)_17_A_50_/PAAc_49_ PIC vesicle at *C*_p_ = 0.1 g/L in water at pH 10 as a function of *f*^+^ at 25 °C.

The mixing ratio of the oppositely charged polymers determines the development of the PIC vesicle [40,41]. The plots of *R*_h_, LSI, and zeta-potential with *f*^+^ at pH 10 were prepared (Figure 3). The *C*_p_ values in the PIC vesicle solutions were kept at 0.1 g/L. However, samples at *f*^+^ = 0 and 1 attributed to anionic PAAc_49_ and cationic P(VS)_17_A_50_, respectively, were prepared at *C*_p_ = 2.0 g/L because *R*_h_ and zeta-potential values could not be obtained because of very low LSI at *C*_p_ = 0.1 g/L. The *R*_h_ and LSI values of the PIC vesicle reached maximum values at *f*^+^ = 0.5, and then the zeta-potential reached 0 mV, suggesting the neutralization of the oppositely charged PAPTAC_50_ block and PAAc_49_. Meanwhile, the zeta-potential values of PAAc_49_ and the PAPTAC_50_ block at *f*^+^ = 0 and 1 were −27.7 and 23.8 mV, respectively, indicating negative and positive charges.

**Figure 4:**
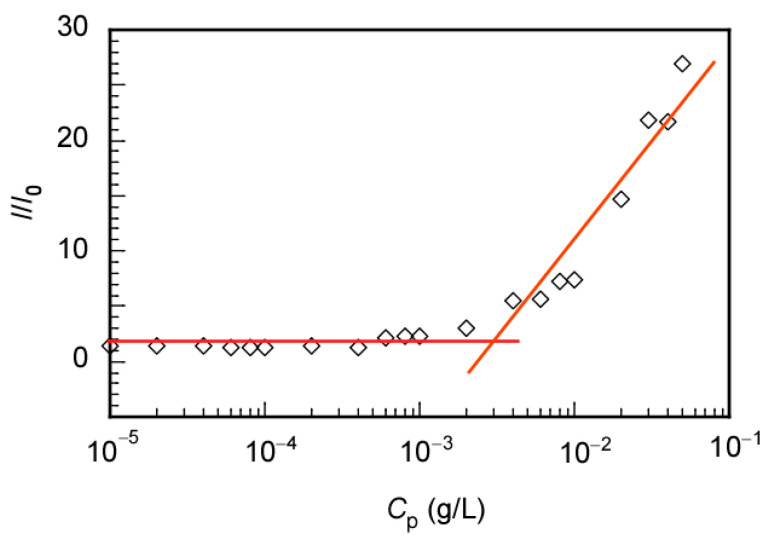
Light scattering intensity ratio (*I*/*I*_0_) for the P(VS)_17_A_50_/PAAc_49_ PIC vesicle in water pH 10 as a function of polymer concentration (*C*_p_). *I* and *I*_0_ are the light scattering intensities of the PIC vesicle solution and solvent, respectively.

The critical micelle concentration (CMC) of the PIC vesicle was determined to certify the thermodynamic equilibrium properties. The LSI of the PIC vesicle aqueous solutions was measured as a function of *C*_p_. The PIC vesicle aqueous solution was prepared by mixing P(VS)_17_A_50_ and PAAc_49_ in water at pH 10 with *f*^+^ = 0.5 at *C*_p_ = 0.1 g/L; the solution was diluted to the desired *C*_p_ using water at pH 10. The CMC for the PIC vesicle in water was determined from the relationship between the LSI ratio (*I*/*I*_0_) and *C*_p_ (Figure 4). *I* and *I*_0_ correspond to the LSIs of the PIC vesicle aqueous solution and water at pH 10, respectively. The crossing point of the linear portions in the low and high *C*_p_ regions was defined as the CMC value, which was 2.47 × 10^−3^ g/L. The defined CMC value implies that the PIC vesicle is in a dynamic equilibrium state. The PIC vesicle is stable above the CMC. The PIC vesicle, however, can disintegrate below the CMC into a small assembly of P(VS)_17_A_50_/PAAc_49_ because of strong electrostatic interactions between them [33,42].

**Figure 5:**
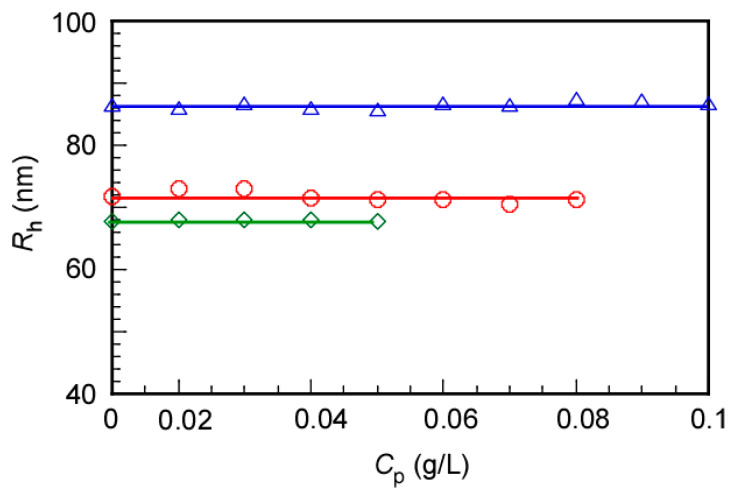
Hydrodynamic radius (*R*_h_) light scattering intensity (LSI) for the P(VS)_17_A_50_/PAAc_49_ PIC vesicle with *f*^+^ = 0.5 in water at pH 10 as a function of polymer concentration after mixing P(VS)_17_A_50_ and PAAc_49_. The PIC vesicle solution at 0.1 (triangle), 0.08 (circle), and 0.05 (diamond) g/L were diluted with pH 10 aqueous solution at 25 °C.

We studied the concentration dependence of the PIC vesicle because the SLS should be measured at different *C*_p_ values to perform the SLS measurements. The P(VS)_17_A_50_ and PAAc_49_ aqueous solutions were separately prepared at *C*_p_ = 0.1, 0.08, and 0.05 g/L. Then, P(VS)_17_A_50_ and PAAc_49_ *C*_p_ were mixed to form the PIC vesicle. The pH value of the aqueous solution was adjusted to 10 and left for one day to obtain an equilibrium state. Subsequently, the PIC vesicle aqueous solutions were diluted by water at pH 10 to adjust the target *C*_p_. The *R*_h_ and LSI for the PIC vesicle were plotted as a function of the *C*_p_ after mixing (Figure 5). The *R*_h_ values for the PIC vesicles at *C*_p_ = 0.1, 0.08, and 0.05 g/L before mixing were 86.6, 71.2, and 67.8 nm, respectively. This indicated that the size of the PIC vesicles increased with an increasing *C*_p_ before mixing since the aggregation number (*N*_agg_) of the PIC vesicle depends on the *C*_p_. When the *C*_p_ increases, the *N*_agg_ increases to increase the size of the PIC vesicle [42]. Furthermore, the increase in the *N*_agg_ was confirmed by of the increase in the LSI value from 1.55 to 3.25 Mcps at *C*_p_ = 0.05 and 0.1 g/L, respectively (Figure S4). When the PIC vesicle was diluted with water at pH 10, the *R*_h_ values remained nearly constant, independent of the *C*_p_. These findings suggest that the size of the PIC vesicle can be controlled by adjusting the *C*_p_ before mixing, and after the PIC vesicleformation they were stable against dilution.

SLS measurements at 25 °C were used to determine further properties of the polymers and PIC vesicle, such as evident weight-average molecular weight (*M*_w_(SLS)), and radius of gyration (*R*_g_) (Figure S5). The RI increment (d*n*/d*C*_p_) values for all samples were also separately estimated. *N*_agg_ is the number of polymer chains required to form a PIC vesicle. The PIC vesicle was prepared by mixing P(VS)_17_A_50_ and PAAc_49_ at C_p_ = 0.1 g/L with *f*^+^ = 0.5 at pH 10 then the solution was diluted to 0.025 g/L by water at pH 10. Table 2 indicates the value of *M*_w_(SLS), *N*_agg_, *R*_g_, and *R*_h_ for all samples. The values of *M*_w_(SLS) for P(VS)_17_A_50_ and PAAc_49_ were 1.55 × 10^4^ g/mol and 0.33 × 10^4^ g/mol, respectively, which were close to the *M*_n_(NMR) values estimated from the ^1^H NMR measurements. P(VS)_17_A_50_ and PAAc_49_ had *M*_n_(NMR) values of 1.42 × 10^4^ and 0.35 × 10^4^ g/mol, respectively. The *M*_w_(SLS) for the PIC vesicle was 1.06 × 10^8^ g/mol. The value of *N*_agg_ for the PIC vesicle was estimated by dividing the *M*_w_(SLS) of the PIC vesicle by that of the unimers and was found to be 5640. The *R*_g_/*R*_h_ value for the PIC vesicle was calculated as 1.17, indicating a spherical shape [3,43,44]. The following equation can be used to calculate the density (*d*) of the polymers and PIC vesicles:

(5) $$d=\frac{M_{w}\left(\mathrm{SLS}\right)}{\frac{4}{3}{\pi N}_{A}R_{h}^{3}}$$

The *d* values for P(VS)_17_A_50_, PAAc_49_, and the PIC vesicle were calculated to be 0.033, 0.028, and 0.064 g/cm^3^, respectively. The *d* value for the PIC vesicle was larger than those for P(VS)_17_A_50_ and PAAc_49_, suggesting that the polymer chains in the PIC vesicle are more densely packed than those of the unimers.

**Figure 6:**
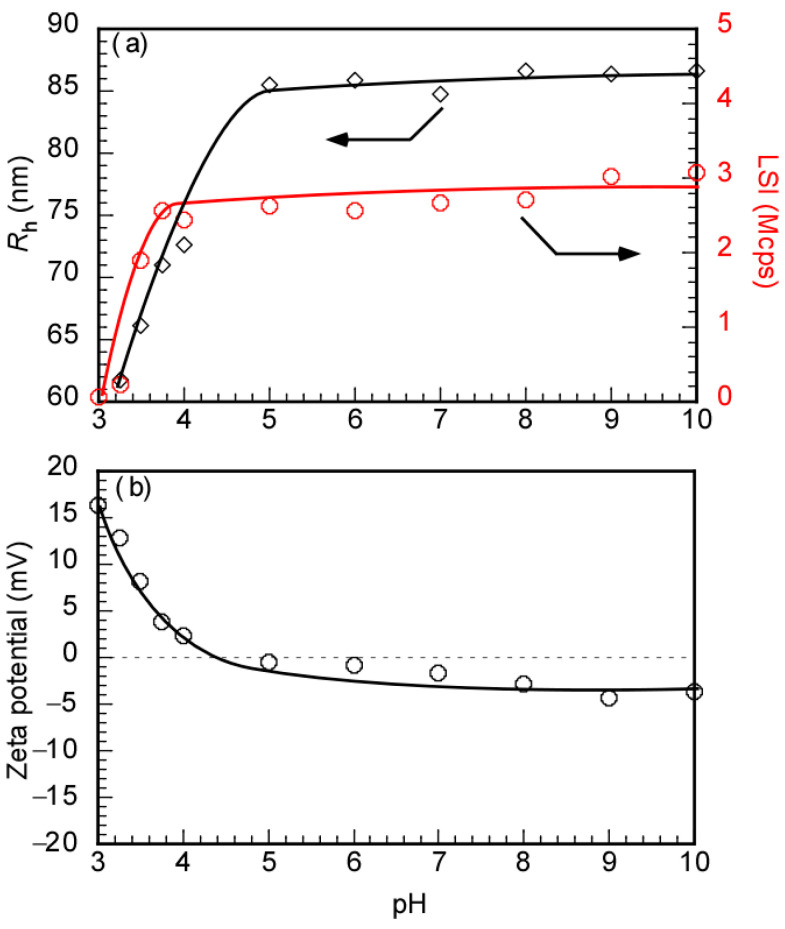
(**a**) Hydrodynamic radius (*R*_h_, diamond) and LSI (circle), and (**b**) zeta-potential for the P(VS)_17_A_50_/PAAc_49_ PIC vesicle with *f*^+^ = 0.5 at *C*_p_ = 0.1 g/L in water as a function of pH at 25 °C.

The pendant carboxylic groups in PAAc_49_ are deprotonated under basic conditions. Therefore, the pH of the solution is an important factor in the PIC vesicle formation. To understand the effect of the pH value on the PIC vesicle, the *R*_h_ and LSI were measured as a function of the pH values. Initially, the PIC vesicle aqueous solution was prepared in water at *C*_p_ = 0.1 g/L with *f*^+^ = 0.5, then adjusted to the appropriate pH using NaOH and HCl aqueous solutions. The *R*_h_, LSI, and zeta-potential values were plotted against the pH. By increasing pH from 3 to 5, the *R*_h_ of the PIC vesicle increased from 60.9 to 85.5 nm, then remained at ca. 86 nm above pH 5 (Figure 6a). The LSI value of the PIC vesicle showed the same tendency as that of the *R*_h_. The acid dissociation constant (p*K*_a_) of PAAc is about 4.5 [45,46,47]. At pH above 4.5 of the PAAc_49_ aqueous solutions, the pendant carboxyl groups were deprotonated to generate anionic charges. The anionic PAAc_49_ with pendant carboxylate anions can interact with the cationic PAPTAC block in P(VS)_17_A_50_ to form the PIC vesicle through electrostatic interactions. This was supported by the zeta-potential value of the PIC vesicle aqueous solutions. At pH < 5, the zeta-potential of the PIC vesicle showed a positive value which can be attributed to a charge of the cationic PAPTAC block in P(VS)_17_A_50_ with protonated PAAc_49_ (Figure 6b). After increasing the pH value, at 5 ≤ pH the zeta-potential reached nearly 0 mV. It implied that the PIC vesicle formation was affected by the pH value, which means that the PIC vesicle can be formed above pH 5 and collapse below pH 5. The pH influence on the association behavior of the PIC vesicle is attributed to the pH-responsive property of PAAc_49_. The conformation of PAAc in water was reported, and a reversible transition from the coil to the globule dependence of the pH was caused by deprotonation of the carboxylic group. At low pH, PAAc was a compact globular shape. When the media of the solution became baser, PAAc expanded into an open coil shape [47,48]. Furthermore, the *R*_h_ and LSI values for P(VS)_17_A_50_ were constant at ca. 6 nm and 70 kcps, respectively, at all pH values ranging from 3 to 10 (Figure S6), suggesting that P(VS)_17_A_50_ was not affected by pH. The small *R*_h_ and LSI values indicated that the polymer was in the unimer state. The zeta-potential values of P(VS)_17_A_50_ and PAAc_49_ were measured as a function of pH (Figure S7). The zeta-potential for P(VS)_17_A_50_ stayed constant at ca. 17 mV, indicating that the polymer always gives a positive charge independent of the solution’s pH. However, the zeta-potential of PAAc_49_ decreased from pH 3 to 5, then remained constant from pH 6. The p*K*_a_ for PAAc_49_ was 4.5; therefore, from pH 5, the PAAc_49_ deprotonated and indicated a negative charge.

**Figure 7:**
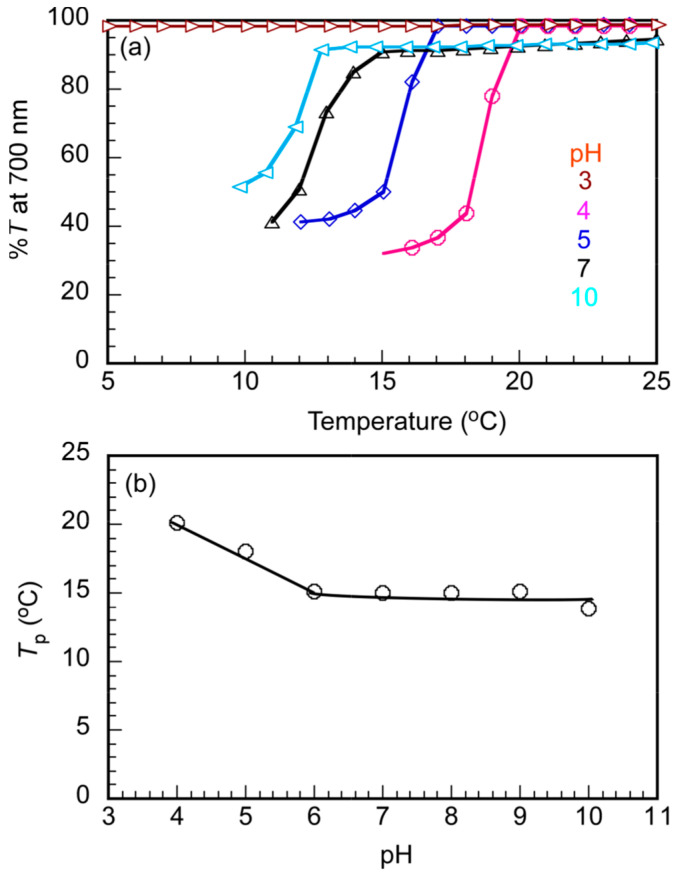
(**a**) Percent transmittance (%*T*) at 700 nm for the P(VS)_17_A_50_/PAAc_49_ PIC vesicle aqueous solution with *f*^+^ = 0.5 as a function of temperature upon the cooling process at different pH at *C*_p_ = 0.1 g/L, and (**b**) pH dependence of the phase transition temperature (*T*_p_) for the PIC vesicle aqueous solution with *f*^+^ = 0.5 at *C*_p_ = 0.1 g/L.

The ^1^H NMR spectra for the PIC vesicle suggested the A_50_/PAAc_49_ PIC membrane was covered with P(VS)_17_ amphoteric random copolymer shells. P(VS)_17_ showed the UCST behavior in aqueous solutions (Figure S8). Therefore, the PIC vesicle was expected to exhibit the UCST behavior owing to the P(VS)_17_ shells. The percentage transmittance (%*T*) of the PIC vesicle was plotted against temperature upon the cooling process indicating the UCST behavior of the PIC vesicle. The temperature dependences investigated the effect of pH on the UCST behavior on %*T* at different pH conditions ranging from pH 3 to 10 (Figure 7). The PIC vesicle was disrupted below pH 5 owing to protonation of the pendant carboxyl groups in PAAc_49_ (Figure 6). However, the aqueous solutions showed the UCST behavior excepted at pH 3. The phase transition temperature (*T*_p_) of the PIC vesicle decreased from pH 4 to 6, then remained at 15 °C from pH 6. The UCST behavior of the PIC vesicle was affected by pH.

**Figure 8:**
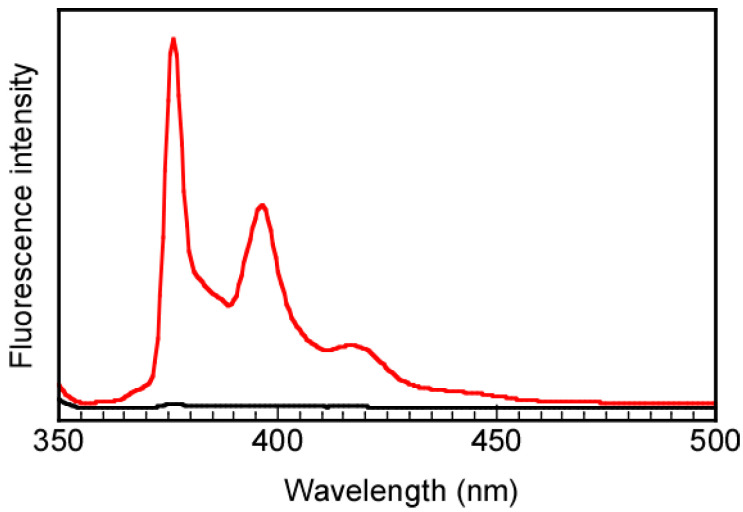
Fluorescence spectra for PEG-Py after dialysis against water at pH 10 for 10 days excited at 334 nm in the presence (—) and absence (—) of the PIC vesicle.

We have studied incorporating a hydrophilic noncharged guest molecule, PEG-Py, into the PIC vesicles. The hydrophilic drugs can be encapsulated inside the PIC vesicle thanks to the inner water phase. Herein, P(VS)_17_A_50_ and PAAc_49_ solutions containing PEG-Py were mixed to form the PIC vesicle. The PEG-Py molecules which could not be incorporated into the PIC vesicle were removed by dialysis against pure water at pH 10. In comparison, a blank experiment was conducted, and the PEG-Py aqueous solution in the absence of the PIC vesicle was dialyzed with the same procedure. After dialysis for 20 days, fluorescence spectroscopy for PEG-Py in the presence and absence of the PIC aggregate was measured (Figure 8). The maximum emission wavelengths at 376 and 397 nm for PEG-Py were observed in the PIC vesicle solution, suggesting that the PIC vesicles can encapsulate PEG-Py. Hydrophobic pyrene molecules associate to produce excimer emission with a peak at 480 nm. PEG-Py can dissolve in water without interpolymer association in this study because no excimer emission was observed from PEG-Py fluorescence. In contrast, no signal of PEG-Py was detected from the reference solution in the absence of the PIC vesicle owing to the removal of the small PEG-Py molecules after dialysis membrane with a pore size of 10 nm. These findings suggested the PIC aggregate might be a vesicle structure because it can encapsulate hydrophilic PEG-Py. From the calibration curve of PEG-Py (Figure S9) and the fluorescence intensity, the weight of PEG-Py encapsulated into a PIC vesicle was estimated to be 0.0012 g. The LE and LC were calculated to be 12% and 1.2%, respectively, from Equations (1) and (2).

**Figure 9:**
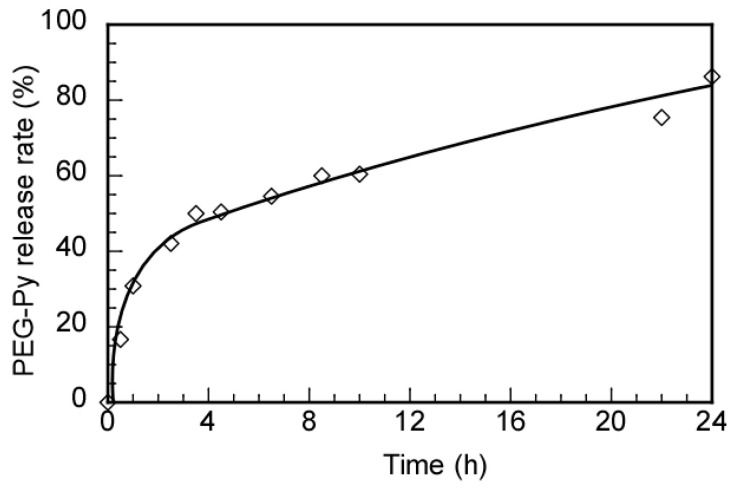
Release profiles of the encapsulated PEG-Py from the PIC vesicle.

Using an HCl aqueous solution, the PIC vesicle encapsulating PEG-Py at pH 10 was adjusted to pH 3. A dialysis bag with a 10-nm pore size was used to dialyze the solution with an aqueous solution at pH 3. The solution sample inside the dialysis bag was taken out at varying times to measure the fluorescence intensities to calculate the release rate of the encapsulated PEG-Py from the PIC vesicle (Figure 9). After 24 h, the release amount of PEG-Py at pH 3 was 86.2%. Noncharged hydrophilic guest molecules can be encapsulated into the PIC vesicle at pH 10 and released at pH 3 owing to the PIC vesicle’s disruption. The release rate of the encapsulated guest molecule may be changed above and below the UCST of the P(VS)_17_ shell. 

## 4. Conclusions

P(VS)_17_A_50_, a cationic diblock copolymer, and an anionic homopolymer, PAAc_49_, were successfully synthesized via a RAFT polymerization with a well-controlled structure. The pH- and thermo-responsive P(VS)_17_A_50_/PAAc_49_ PIC vesicle was prepared by mixing P(VS)_17_A_50_ and PAAc_49_ in basic aqueous solutions at room temperature due to electrostatic interactions. The *R*_h_ and LSI values of the PIC vesicle showed maximum values at *f*^+^ = 0.5, whereas the zeta-potential was near 0 mV. At low pH, the PIC vesicle disintegrated. The spherical shape with a vesicle structure was confirmed by TEM. The *R*_g_/*R*_h_ ratio obtained from SLS and DLS measurements showed a value close to 1, indicating the spherical shape of the PIC vesicle. The thermo-responsive P(VS)_17_ shells caused UCST behavior in the PIC vesicle. The thermo-responsive behavior of the PIC vesicle was affected by the pH of the solution. The PIC vesicle can encapsulate water-soluble nonionic guest molecules at a high pH and release them under acidic conditions, implying that it could be used as a DDS material. The interaction of the PIC vesicle with the cell can be controlled by temperature because the surface hydrophobicity increases with decreasing temperature. Therefore, it is expected that the PIC vesicle can be taken in the cell at a low temperature below the UCS of the P(VS)_17_ shells. It is known that the pH around the cancer tissue is low. The encapsulated hydrophilic anticancer drugs can be released under acidic conditions from the P(VS)_17_A_50_/PAAc_49_ PIC vesicle.

## Figures and Tables

**Table 1:** Degree of polymerization (DP), number-average molecular weight (*M*_n_), and polydispersity index (*M*_w_/*M*_n_).

Sample	DP (theo)	DP (NMR)	*M*_n_(theo) ^1^ × 10^−4^ (g/mol)	*M*_n_(NMR) ^2^ × 10^−4^ (g/mol)	*M*_n_(GPC) ^3^ × 10^−4^ (g/mol)	*M*_w_/*M*_n_
P(VS)_17_	17	-	0.38	-	-	-
P(VS)_17_A_50_	49	50	1.40	1.41	5.70	1.04
PAAc_49_	36	49	0.26	0.34	1.40	1.07

^1^ Calculated from Equation (4) ^2^ Obtained from ^1^H NMR ^3^ Obtained from GPC.

**Table 2:** Dynamic and static light scattering data for P(VS)_17_A_50_, PAAc_49_, and the P(VS)_17_A_50_/PAAc_49_ PIC vesicle.

Sample	*M*_w_(SLS) × 10^−4^ (g/mol)	*N*_agg_	*R*_g_ (nm)	*R*_h_ (nm)	*R*_g_/*R*_h_	d*n*/d*C*_p_	*d*^1^ (g/cm^3^)
P(VS)_17_A_50_	1.55	1	-	5.7	-	0.1323	0.033
PAAc_49_	0.33	1	-	5.3	-	0.2278	0.028
PIC vesicle	10,600	5640	101	86.6	1.17	0.0736	0.064

^1^ Calculated from Equation (5).

## Data Availability

The data used to support the findings of this study are available from the corresponding authors upon request.

## Supplementary Materials

The following supporting information can be downloaded at: https://www.mdpi.com/article/10.3390/polym14091659/s1, Figure S1. ^1^H NMR spectrum for P(VBTAC/NaSS)_17_ in D_2_O containing 1.2-M NaCl at 70 °C; Figure S2. Gel-permeation chromatography (GPC) elution curves for (**a**) P(VS)_17_A_50_ and (**b**) PAAc_49_ monitored with refractive index (RI) detector working at 40 °C; Figure S3. FTIR spectrum for P(VS)_17_A_50_; Figure S4. Light scattering intensity (LSI) for the P(VS)_17_A_50_/PAAc_49_ PIC vesicles with *f*^+^ = 0.5 in water at pH 10 as a function of polymer concentration after mixing P(VS)_17_A_50_ and PAAc_49._ At 25 °C the PIC aggregates solution at 0.1 (triangle), 0.08 (circle), and 0.05 (diamond) g/L were diluted with pH 10 aqueous solution; Figure S5. Typical examples of Zimm plots for (**a**) P(VS)_17_, (**b**) PAAc_49_, and (**c**) the P(VS)_17_A_50_/PAAc_49_ PIC vesicle with *f*^+^ = 0.5 in aqueous solutions at pH 10; Figure S6. Hydrodynamic radius (*R*_h_, diamond) and light scattering intensity (LSI, circle) for P(VS)_17_A_50_ at *C*_p_ = 2.0 g/L in water as a function of pH at 25 °C; Figure S7. Zeta-potential for P(VS)_17_A_50_ (circle) and PAAc_49_ (triangle) in water at *C*_p_ = 2.0 g/L as a function of pH at 25 °C; Figure S8. Percent transmittance (%*T*) at 700 nm for the 0.1-M NaCl aqueous P(VS)_17_ solution at *C*_p_ = 1.0 g/L as a function of temperature upon the heating and cooling processes; Figure S9. (**a**) Fluorescence spectra for PEG-Py in water at pH 10 excited at 334 nm at various PEG-Py concentrations ([PEG-Py]) and (**b**) a calibration curve of fluorescence intensity at 376 nm using [PEG-Py] in water at pH 10.
