# Supplementary material for: pH- and Thermo-Responsive Water-Soluble Smart Polyion Complex (PIC) Vesicle with Polyampholyte Shells
Source: Polymers (Basel). 2022 Apr 20;14(9):1659. doi: 10.3390/polym14091659 (PMC9099632; doi:10.3390/polym14091659)
Supplement: Supplementary file 1 [file polymers-14-01659-s001.zip › polymers-1697961-supplementary.pdf]

Supplementary Materials

pH- and Thermo-Responsive Water-Soluble Smart Polyion Complex (PIC) Vesicle with Polyampholyte Shells

Thu Thao Pham[1], Tien Duc Pham[2], and Shin-ichi Yusa[1*]

[1]Department of Applied Chemistry, Graduate School of Engineering, University of Hyogo, 2167 Shosha, Himeji, Hyogo 671-2280, Japan

[2]Faculty of Chemistry, University of Science, Vietnam National University, Hanoi, 19 Le Thanh Tong, Hoan Kiem, Hanoi 100000, Vietnam

*Corresponding author

Shin-ichi Yusa

yusa@eng.u-hyogo.ac.jp

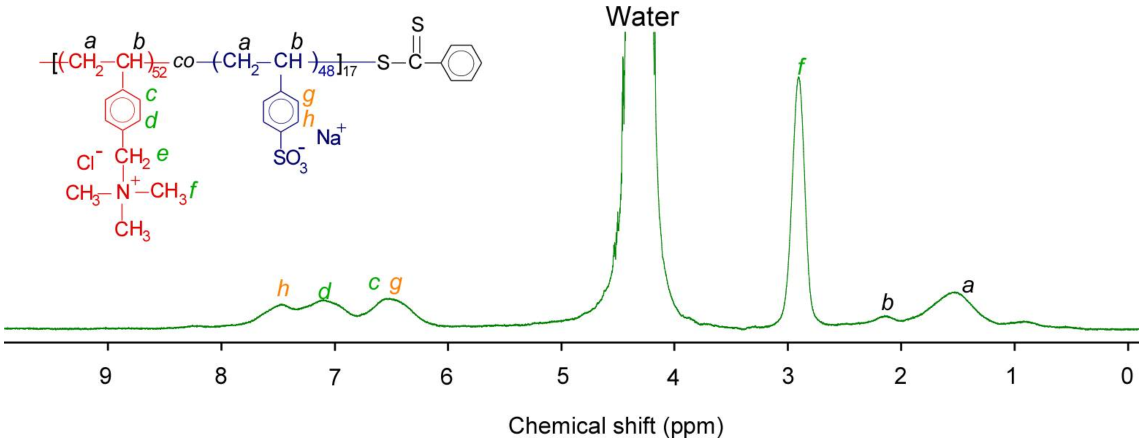

Figure S1. ^{1}H NMR spectrum for P(VBTAC/NaSS)$_{17}$ in D_2O containing 1.2-M NaCl at 70 °C.

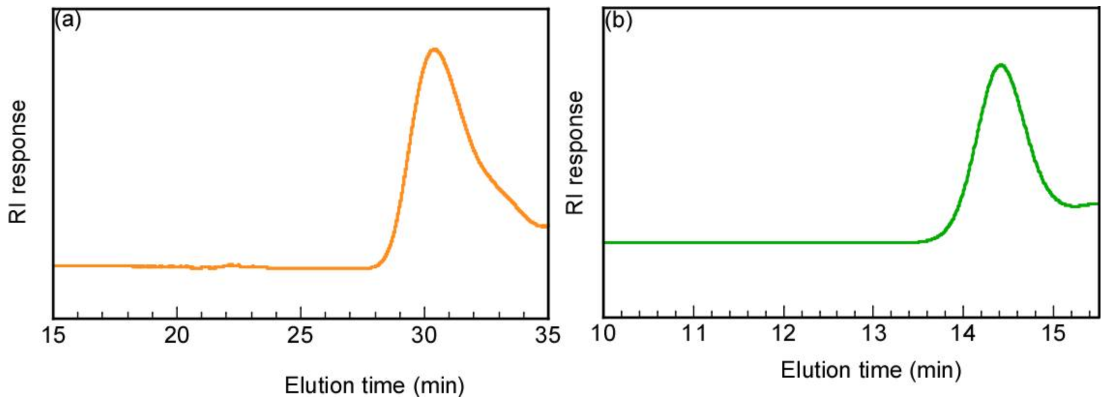

Figure S2. Gel-permeation chromatography (GPC) elution curves for (a) P(VS)$_{17}$A$_{50}$ and (b) PAAc$_{49}$ monitored with refractive index (RI) detector working at 40 °C.

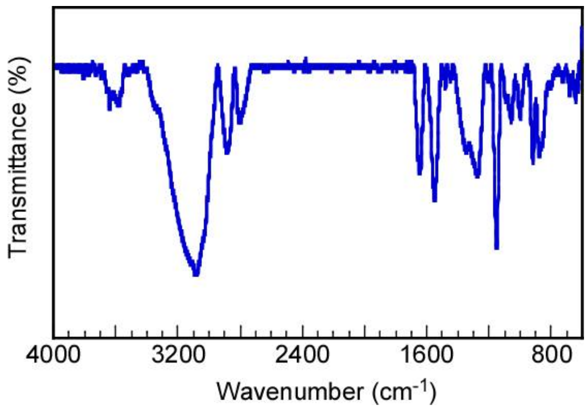

Figure S3. FTIR spectrum for $P(VS)_{17}A_{50}$.

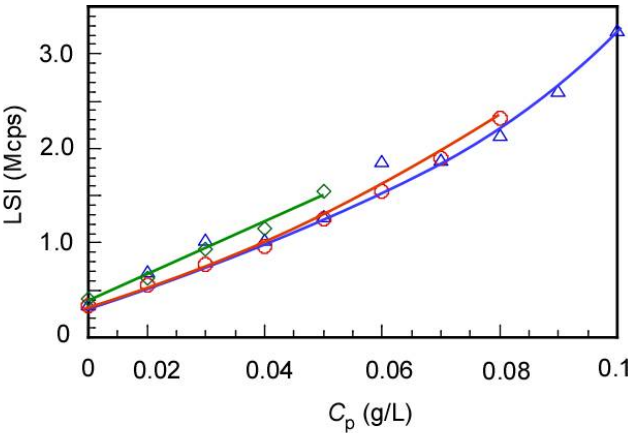

Figure S4. Light scattering intensity (LSI) for the $P(VS)_{17}A_{50}$/$PAAc_{49}$ PIC vesicles with $f^{+} = 0.5$ in water at pH 10 as a function of polymer concentration after mixing $P(VS)_{17}A_{50}$ and $PAAc_{49}$. At 25 °C the PIC aggregates solution at 0.1 (triangle), 0.08 (circle), and 0.05 (diamond) g/L were diluted with pH 10 aqueous solution.

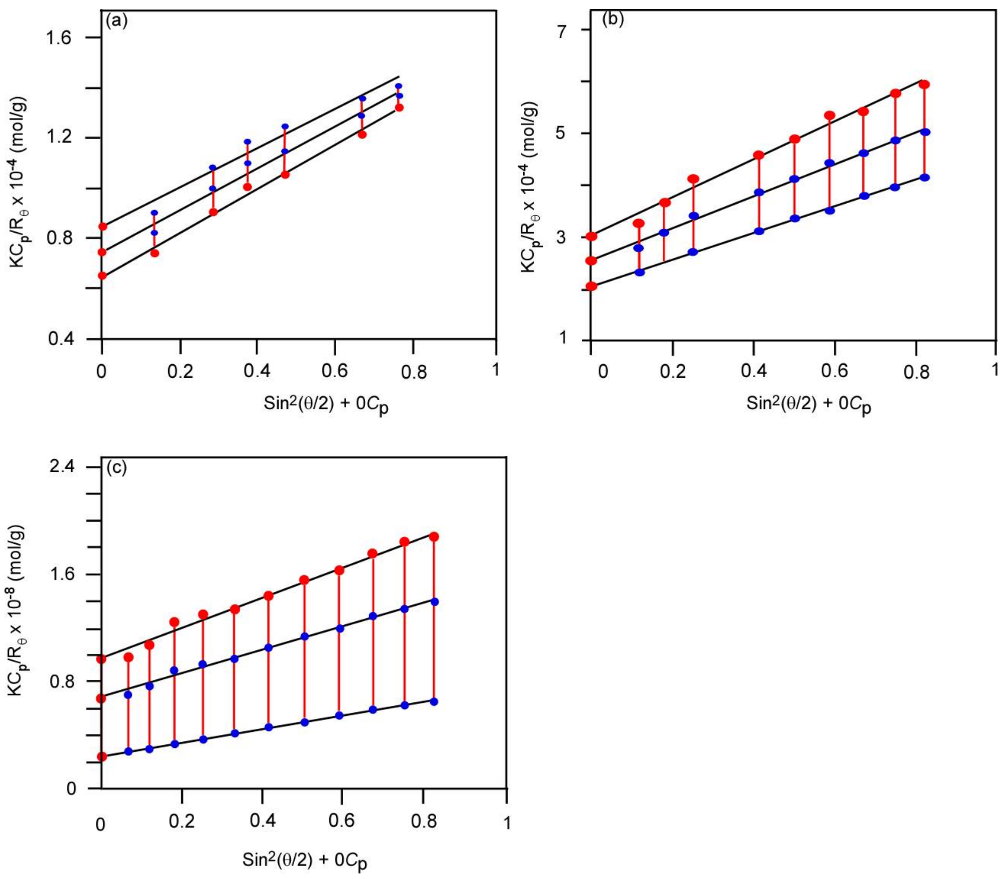

Figure S5. Typical examples of Zimm plots for (a) $P(VS)_{17}$, (b) $PAAc_{49}$, and (c) the $P(VS)_{17}A_{50}/PAAc_{49}$ PIC vesicle with $f^+ = 0.5$ in aqueous solutions at pH 10.

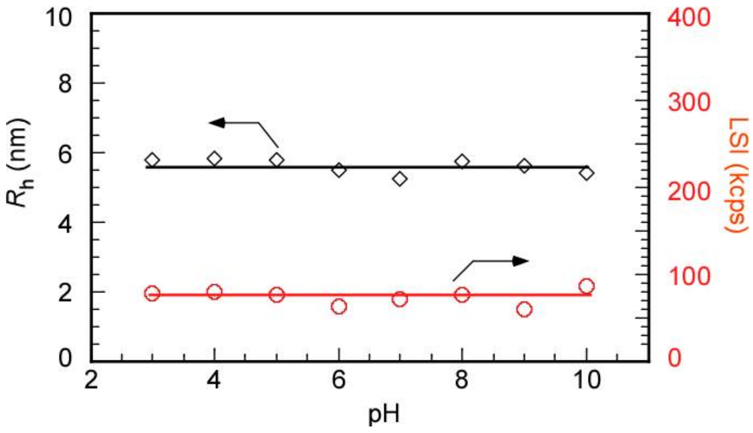

Figure S6. Hydrodynamic radius (R_h, diamond) and light scattering intensity (LSI, circle) for $P(VS)_{17}A_{50}$ at $C_p = 2.0$ g/L in water as a function of pH at 25 °C.

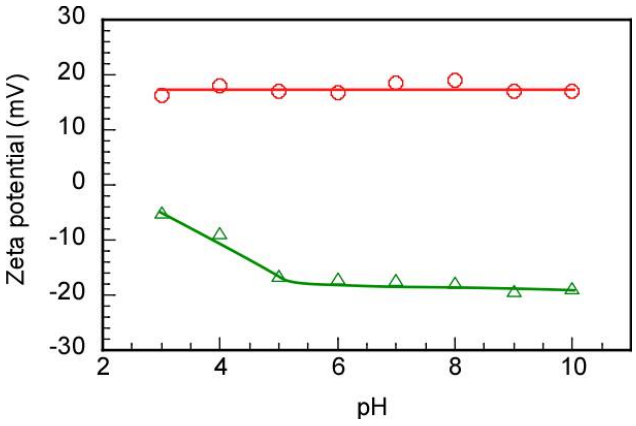

Figure S7. Zeta-potential for $P(VS)_{17}A_{50}$ (circle) and $PAAc_{49}$ (triangle) in water at C_p = 2.0 g/L as a function of pH at 25 °C.

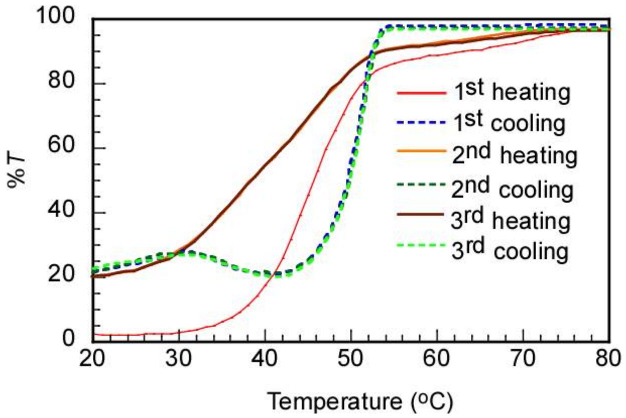

Figure S8. Percent transmittance (%*T*) at 700 nm for the 0.1-M NaCl aqueous $P(VS)_{17}$ solution at C_p = 1.0 g/L as a function of temperature upon the heating and cooling processes.

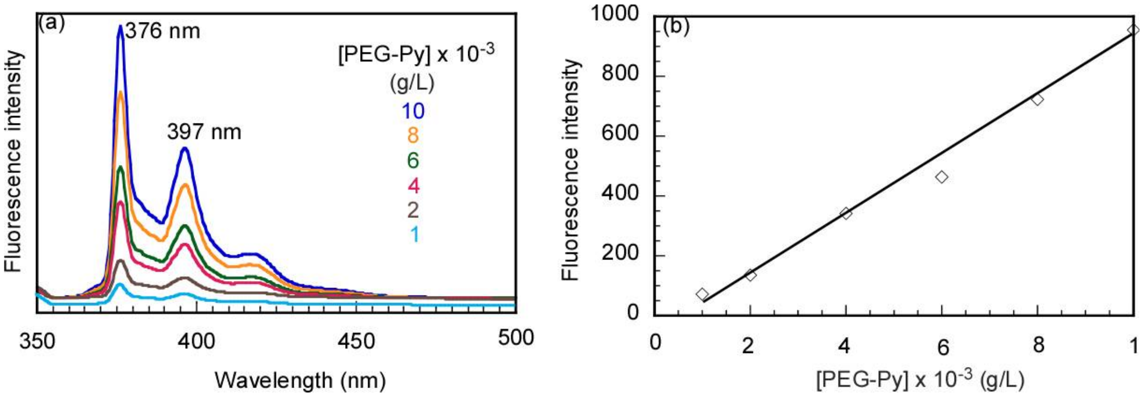

Figure S9. (a) Fluorescence spectra for PEG-Py in water at pH 10 excited at 334 nm at various PEG-Py concentrations ([PEG-Py]) and (b) a calibration curve of fluorescence intensity at 376 nm using [PEG-Py] in water at pH 10.
